# Author Correction: Mathematical modelling of SARS-CoV-2 variant outbreaks reveals their probability of extinction

**DOI:** 10.1038/s41598-022-19152-1

**Published:** 2022-08-30

**Authors:** Henrik Schiøler, Torben Knudsen, Rasmus Froberg Brøndum, Jakob Stoustrup, Martin Bøgsted

**Affiliations:** 1 Department of Electronic Systems, Aalborg University, Aalborg, Denmark; 2 Department of Clinical Medicine, Aalborg University, Aalborg, Denmark; 3 Department of Haematology, Aalborg University Hospital, Aalborg, Denmark

Correction to: *Scientific Reports* 10.1038/s41598-021-04108-8, published online 30 December 2021

The original version of this Article contained an error in the Discussion section, where

“However, at the time of writing (March 1, 2021), the variant has not emerged, so the probability of extinction is now well above 0.95%.”

now reads:

“However, at the time of writing (March 1, 2021), the variant has not emerged, so the probability of extinction is now well above 95%.”

The original Article has been corrected.

